# The Cradle to Prison Pipeline: An American Health Crisis

**Published:** 2007-06-15

**Authors:** Marian Wright Edelman

**Affiliations:** Children’s Defense Fund

Suppose that during the next decade, a quarter of all the children born in New York, North Carolina, Texas, Colorado, Ohio, and Pennsylvania were infected by a virulent new strain of polio or tuberculosis sometime during their youth. Clearly, our response to a health crisis affecting that many children would be to mobilize the nation's vast public health resources. Medical laboratories would operate around the clock to develop new vaccines.

Unfortunately, an infection akin to this hypothetical tragedy is actually coursing through African American and Latino communities across the nation. I'm not referring to a virus such as HIV/AIDS or a hazardous bacterium. I'm talking about the criminalization of poor children and children from minority races who enter what the Children's Defense Fund (CDF) identified as America's Cradle to Prison Pipeline. Together, African Americans and Latinos comprise a segment of the U.S. population equal to that of the six states I mentioned earlier. Like the victims of a crippling or wasting disease, once drawn into the prison pipeline, massive numbers of young people lose their opportunity to live happy, productive lives, not because of festering microbes but because of years spent behind bars.

Through its Cradle to Prison Pipeline initiative, the Children's Defense Fund has studied the grim effects of being trapped in a criminalizing environment from which the obstacles to escape are formidable. The Cradle to Prison Pipeline consists of a complex array of social and economic factors as well as political choices that converge to reduce the odds that poor children — especially poor black and Latino children — will grow up to become productive adults. These factors include limited access to health care (including mental health care), underperforming schools, broken child welfare and juvenile justice systems, and a toxic youth culture that praises pimps and glorifies violence.

Hardened by long terms of incarceration, released criminalized youngsters return to communities that are ill equipped to reintegrate them positively. Outcast and unemployed, they become the teachers and role models for a new crop of youngsters pushed onto the streets of America's most depressed neighborhoods. This cycle of infection makes the Cradle to Prison Pipeline one of the most damaging health problems in America today.

A major factor in determining whether a child enters the prison pipeline is access to health care. Currently, nine million children in America are without health insurance (1). Among low-income communities, there is a high incidence of teen pregnancy and low-birthweight babies (1). Physical and mental developmental delays among young children are commonly left undiagnosed and often go untreated (2,3). Unlike the children from affluent families, children from low-income families rarely have access to institutions that can intervene and address their health problems (2,3).

Few public schools in economically depressed neighborhoods have the resources to recognize health issues such as dyslexia, attention deficit disorder, hyperactivity disorder, or post-traumatic stress disorder and then to provide counseling and therapy for children with these disorders (1). Instead, their behavior is more often perceived as insubordinate or disruptive than it is recognized as symptomatic of a disorder or of the environment in which these children live (1,4). In these cases, zero-tolerance disciplinary standards are frequently applied, and thousands of students are expelled and even arrested for subjectively defined behaviors such as "disorderly conduct" and "malicious mischief" (5).

We must dismantle the Cradle to Prison Pipeline now because all children are sacred. What is required are collaborative efforts at the community, municipal, and state levels. To start with, we should demand the passage of legislation that would guarantee health care, including mental health care, to all children.

We need new investment to support proven community health delivery programs such as the National Campaign to Prevent Teen Pregnancy, which promotes community and school programs focused on delaying sexual activity (6), and the Nurse-Family Partnership, which supplies nurses for home visits to low-income, first-time mothers through their pregnancies and for two years after they give birth (7). Other valuable programs provide early intervention in cases of family violence (8). A healthy child is an empowered child. Communities should strive to replicate model umbrella programs that mentor and empower children such as the Harlem Children's Zone (9), the Boston TenPoint Coalition (10), and the CDF Freedom Schools program (11).

The effects of the Cradle to Prison Pipeline constitute a scourge of epidemic proportions. We must act to dismantle the prison pipeline now. We fail at our peril. The future of our nation is at stake.
